# Discovery and pharmacological study of a novel diuretic

**DOI:** 10.1186/1479-5876-10-S2-A63

**Published:** 2012-10-17

**Authors:** Baoxue Yang, Fei Li, Hong Zhou, Tianluo Lei

**Affiliations:** 1 Department of Pharmacology, School of Basic Medical Sciences, Peking University, Beijing, 100191, China

## 

Diuretics are used widely to raise renal salt and water clearance in a variety of conditions, such as oedema, as well in non-edematous states such as hypertension, which can reduce morbidity and mortality of cardiovascular and cerebrovascular diseases, especially the frequency of stroke and congestive heart failure. However, long-term use of conventional diuretics has several adverse effects including electrolyte disorders, hyperuricemia, hyperlipidemia, and glucose tolerance decrease. Electrolyte abnormalities can induce cardiac arrythmias and sudden death. Therefore, discovering a new diuretic that does not cause electrolyte disturbance becomes a hot issue. Phenotype analysis of knockout mice lacking urea transporter UT-B or various UT-A isoforms has provided evidence for the involvement of UTs in the urinary concentrating mechanism. Functional deletion of UT-B or UT-A isoforms markedly caused polyuria and urea selective low urine concentrating ability. However, deletion of UT-B or UT-As did not affect GFR and clearance rate of the principal solutes (Na^+^, K^+^, Cl^-^) in urine except for urea. Therefore, we suggested a hypothesis that UT inhibitors might be novel diuretics to excrete water without disturbing electrolyte metabolism.

Present study discovered a potent small-molecular urea transporter inhibitor, UT-A4, using an erythrocyte osmotic lysis assay. Stopped flow light scattering experiment, a classical assay for measuring water and urea permeability, confirmed that UT-A4 reversibly inhibited UT-B activity. The experiments also showed that UT-A4 targeted the intracellular part of UT-B protein and had the same inhibition activity on influx and efflux of urea across membrane. UT_inh_-14 has inhibition activity on human, rabbit, rat and mouse UT-B. We used rats as an *in vivo* test model for determining the diuretic activity of UT-A4. Interestingly, UT-A4 caused dose-dependent polyuria, low urinary osmolality and urea concentration in rats. 18-h water deprivation raised urine concentrating ability in rats with or without UT-A4 treatment. However, urine osmolality and urea concentration remained significantly less in UT-A4 treated rats than that in control rats, except of unchanged non-urea solutes. Osmolality and urea concentration was significantly decreased in inner medullary tissue of UT_inh_-14 treated rats, but not in HCTZ treated rats. All these results suggest that UT_inh_-14 caused urea selective diuresis.

The excretion of osmoles, urea and non-urea solutes had no significant difference between control and UT_inh_-14 treated rats. However, HCTZ treated rats had significant higher excretion of osmole and non-urea solutes than control and UT_inh_-14 treated rats, which made lower blood Na^+^, K^+^ and Cl^-^. These results indicate that UT-A4 is a selective UT inhibitor and has urea selective diuretic activity without disturbing excretion of electrolytes. It might have potential value on drug discovery as a new diuretic without electrolyte imbalance and metabolic disorder. It might also be used as a tool drug to study the physiological roles of UTs in big animal models.

